# Factors associated with post-mortem notification of tuberculosis cases in Brazil, 2014

**DOI:** 10.1590/0102-311XEN301521

**Published:** 2023-06-26

**Authors:** Ursila Manga Aridja, Marli Souza Rocha, Patrícia Bartholomay, Daniele Maria Pelissari, Daiane Alves da Silva, Katia Crestine Poças, Elisabeth Carmen Duarte

**Affiliations:** 1 Universidade de Brasília, Brasília, Brasil.; 2 Secretaria de Vigilância em Saúde, Ministério da Saúde, Brasília, Brasil.; 3 Faculdade de Saúde Pública, Universidade de São Paulo, São Paulo, Brasil.

**Keywords:** Tuberculosis, Notification, Information Systems, Tuberculose, Notificação, Sistemas de Informação, Tuberculosis, Notificación, Sistemas de Información

## Abstract

According to the World Health Organization (WHO), 1.6 million deaths and 10.6 million cases of tuberculosis (TB) were reported worldwide in 2021. If treated opportunely with the recommended therapy, 85% of patients with TB are healed. The occurrence of death from TB without prior notification of the disease indicates failure in the timely access to this effective treatment. Therefore, this study aimed to identify TB cases with post-mortem notification in Brazil. This is a nested case-control study using a cohort of new TB cases reported to the Braziliam Information System for Notificable Diseases (SINAN). This study analyzed the following variables: selected characteristics of the individual (gender, age, race/color, education), the municipality (Municipality Human Development Index - M-HDI, poverty rate, size, region, and municipality), health services, and underlying or associated cause of death. Logistic regression was estimated using a hierarchical analysis model. People with TB aged 60 years or older (OR = 1.43), with low educational level (OR = 1.67), and with malnutrition (OR = 5.54), living in municipalities with low M-HDI and medium population size (OR = 1.26), located in the North Region of Brazil (OR = 2.42) had a higher chance of post-mortem notification. Protective factors were HIV-TB coinfection (OR = 0.75), malignant neoplasms (OR = 0.62), and living in cities with broad primary care coverage (OR = 0.79). Vulnerable populations should be prioritized in order to address the obstacles to the access to TB diagnosis and treatment in Brazil.

## Resumo

Segundo a Organização Mundial da Saúde (OMS), estima-se que 1,6 milhão de mortes e 10,6 milhões de casos de tuberculose (TB) ocorreram no mundo em 2021. Quando a doença é oportunamente tratada com o esquema terapêutico recomendado, 85% dos pacientes se curam. A ocorrência de óbito por TB sem notificação anterior denuncia falhas no acesso ao tratamento oportuno e efetivo. Sendo assim, este estudo objetivou caracterizar os casos de TB notificados pós-óbito no Brasil. Trata-se de estudo caso-controle aninhado na coorte de casos novos de TB notificados ao Sistema de Informação de Agravos de Notificação (SINAN). As variáveis analisadas foram: características selecionadas do indivíduo (sexo, idade, raça/cor, escolaridade), do município (Índice de Desenvolvimento Humano Municipal - IDH-M, taxa de pobreza, porte municipal, região e município), dos serviços de saúde e causa básica ou associada de morte. Foi estimada regressão logística respeitando um modelo de análise hierárquico. Pessoas com TB de 60 anos de idade ou mais (OR = 1,43), de baixa escolaridade (OR = 1,67), com desnutrição (OR = 5,54), residentes em municípios com baixo IDH-M, de porte populacional médio (OR = 1,26), na Região Norte (OR = 2,42) apresentaram maior chance de notificação pós-óbito. Fatores protetores foram coinfecção HIV-TB (OR = 0,75), neoplasias malignas (OR = 0,62) e residência em municípios com alta cobertura de atenção básica (OR = 0,79). A priorização das populações vulneráveis é necessária para enfrentar as dificuldades de acesso ao diagnóstico e tratamento da TB no Brasil.

## Resumen

La Organización Mundial de la Salud (OMS) estima que en 2021 se produjeron 1,6 millones de muertes por tuberculosis (TB) y 10,6 millones de casos de esta afección por todo el mundo. Si los pacientes siguen el tratamiento recomendado para la TB, un 85% logran la cura. Las muertes por TB sin notificación previa de caso indican fallas en el acceso a este tratamiento oportuno y efectivo. Por lo tanto, este estudio tuvo como objetivo caracterizar los casos de TB que tuvieron notificación posterior a la muerte en Brasil. Este es un estudio de caso-control anidado dentro de la cohorte de nuevos casos de TB informados al Sistema de Información de Enfermedades de Notificación Obligatoria (SINAN). Las siguientes variables fueron analizadas: características seleccionadas del individuo (sexo, edad, etnia/color, nivel de instrucción) y del municipio (Índice de Desarrollo Humano Municipal -IDH-M, tasa de pobreza, tamaño del municipio, región y municipio), servicios de salud y condiciones y causa de la muerte o su asociación. La regresión logística se estimó desde un modelo de análisis jerárquico. Las personas con TB de 60 años o más (OR = 1,43), con bajo nivel de instrucción (OR = 1,67), con desnutrición (OR = 5,54), residentes en municipios con bajo IDH-M, de tamaño poblacional medio (OR = 1,26) y en la Región Norte (OR = 2,42) tuvieron mayor probabilidad de notificación posterior a la muerte. Los factores protectores fueron la coinfección VIH-TB (OR = 0,75), neoplasias malignas (OR = 0,62) y vivir en ciudades con alta cobertura de atención primaria (OR = 0,79). Es necesario priorizar las poblaciones vulnerables para enfrentar las dificultades de acceso al diagnóstico y tratamiento de la TB en Brasil.

## Introduction

Tuberculosis (TB) is a serious infectious disease, but death from TB can be considered a preventable event in most cases ^1^. For sensitive pulmonary TB, the diagnosis, clinical management, and effective treatment are available at all levels of care from the Brazilian Unified National Health System (SUS), preferably provided in primary health care in the Brazil ^1^. Despite that, 10 million people had TB and 145,000 died from TB worldwide in 2018 ^2^. The Americas represented 3% of these cases and Brazil accounted for 32% of new cases in the region ^2^. It means more than 75,000 new cases and around 4,400 deaths every year in the country ^3^.

Considering the scenario described above, the *End TB* strategy was created in 2014, in line with the Sustainable Development Goals (SDGs) of the World Health Organization (WHO) ^4^. This strategy has three pillars: integrated, patient-centered care and prevention; bold policies and supportive systems with an emphasis on protection of vulnerable populations; and intensified research and innovation ^4^. In order to build effective and comprehensive strategies that address these three pillars, underreporting of cases and deaths from TB and broad access to opportune and effective diagnosis and treatment are challenges to overcome, allowing the achievement of national and international goals ^5^.

A previous descriptive study ^6^ used probabilistic relationship in large health surveillance databases and identified that 2,506 (93%) of all TB cases in Brazil in 2014 were not properly notified on the Brazilian Information System on Notificable Diseases (SINAN) and were only detected at the patient’s death. These cases with post-mortem notification present highly vulnerability and a hypothesis is that a significant number of these patients had no access to the health care network, particularly TB-related care.

Post-mortem notification of TB cases is a complex phenomenon and the factors associated with it must be better understood. Although a study was found in the literature addressing underreported TB deaths in Brazil, it has no study that characterizes the factors associated with post-mortem notification of TB cases at national level; therefore, this is the objective of this study.

## Method

This is a nested case-control study using a cohort of new TB cases reported to surveillance systems in Brazil.

The study population consisted of TB cases (all clinical forms) of individuals aged 15 years or more, who reported their gender in the form, and who died in Brazil in 2014, the reference notification year selected for this study.

Data were obtained from the probabilistic relationship between the database of the SINAN-TB for 2014 and the database of the Brazilian Mortality Information System (SIM) for 2014 and 2015. This procedure was performed by technicians from the Brazilian National Tuberculosis Control Program (PNCT) from the Brazilian Ministry of Health and data were provided to the researchers without patient identification.

SIM notifications that matched one SINAN-TB notification were considered as proper notifications (case notification before death) (controls). SIM notifications that did not match any SINAN-TB notification were considered as post-mortem notifications (cases). The only exception was those notifications found in SIM and SINAN-TB systems and recorded in the SINAN-TB as post-mortem. These were also added to post-mortem notifications (cases).

In SIM, records with TB as the underlying or associated cause were used, as identified in part 1 or 2 of the Death Certificate (DC) - codes A15.0 to A19.0 of the 10th revision of the International Classification of Diseases (ICD-10) ^7^.

The following variables of interest were considered:

(a) Individual characteristics: gender, age in years (15-20, 20-40, 40-60, ≥ 60 years), education [none, 1-11 years, 12 or more years, unknown (ignored or does not applies)], race/color (white and yellow, black and brown, Indigenous, ignored). The white and yellow categories were analyzed together to optimize the statistical power of the analysis.

(b) Municipal characteristics (municipality of residence): Municipal Human Development Index (M-HDI) (low: < 0.6, medium: 0.6-0.7, high: > 0.7, ignored); poverty rate: proportion of individuals with per capita income equal to or less than BRL 140.00 per month ^8^ (in August 2010 BRL currency) (low: < 10, medium: 10-20, high: > 20-45, very high: > 45, ignored); population size (small: < 20,000 inhabitants, medium: 20,000-100,000 inhabitants, large > 100,000 inhabitants, ignored); region of the municipality (the five Brazilian regions); municipality of residence (the eight cities with the highest numbers of post-mortem notifications of TB were considered separately, the other cities were combined).

The variables M-HDI, poverty rate, and population size - and respective categorizations - were obtained from the Brazilian Institute of Geography and Statistics (IBGE) ^8^ and the United Nations Development Program (UNDP 2013) ^9^.

(c) Causes of death and the main diseases mentioned in the DC as the underlying or associated cause. The most frequent causes of death and/or causes that require continued attention from health services, such as non-transmissible chronic diseases, were selected.

(d) Characteristics of health services: legal nature of the health service where the death occurred (public, private, non-profit service, ignored); coverage of the Family Health Strategy (FHS) (low: < 50%, medium: 50-75%, high: > 75%); and primary health care coverage (low: < 50%, medium: 50-75%, high: > 75); medical care for the disease that caused the death as notified in the D (yes, no, ignored).

Data related to the variables of FHS and primary health care coverage were obtained from Brazilian Health Informatics Department (DATASUS) ^10^, as well as the type of health establishment on Brazilian National Registry of Health Facilities (CNES) ^10^.

The technical team from the Brazilian Ministry of Health performed the probabilistic relationship using the Reclink III ^11^ software with a routine of multiple steps, each of them using a certain blocking key. A probabilistic relationship has a standardization step, whose objective is to harmonize the files for later use. The next step is called “relationship” and consists of two processes: blocking and pairing of records, which help optimize the comparison process by dividing the databases into logical blocks, and build scores based on the blocking strategy being used. The relationship parameters were estimated by applying Expectation-Maximization (EM) algorithms. The last step refers to data relationship and allows the creation of a new file from two related files. The pairs considered ‘true’ are identified according to a defined score by checking the full names of the person, the mother, and the birth date. At each blocking step, a manual review was performed. Doubtful records were classified as “non-pairs”.

A crude and multivariate analysis was performed to check for associations between the variables of interest and the outcome, using unconditional logistic regression. In the crude analysis, associations between independent variables with the outcome of “post-mortem notification” (yes, no) were evaluated; variables with p ≤ 0.20 were eligible for the multivariate analysis.

In the next step, a hierarchical analysis model was used. Variables of individual characteristics were included in the distal hierarchical level and were adjusted among themselves; municipal characteristics and causes of death were the variables included in the intermediate (medial) hierarchical level and were adjusted among themselves and by statistically significant variables (p < 0.05) of the distal hierarchical level; the variables referring to the characteristics of the health services were included in the proximal hierarchical level and were adjusted among themselves and by significant variables of the intermediate and distal hierarchical levels. At every level of the multivariate analysis, only those variables that significantly helped explain the outcome (p < 0.05) were maintained, using the manual stepwise backwards strategy. The result was the combination of these different final models adjusted by more distal hierarchical levels in relation to the analysis. The analyses were performed using Microsoft Office Excel 2013 (https://products.office.com/) and Stata, versão 11.0 (https://www.stata.com).

This study observed the ethical standards for research with humans. As we exclusively used secondary non-nominal data of public access, it did not require approval by a Research Ethics Committee.

## Results

**Figure 1:**
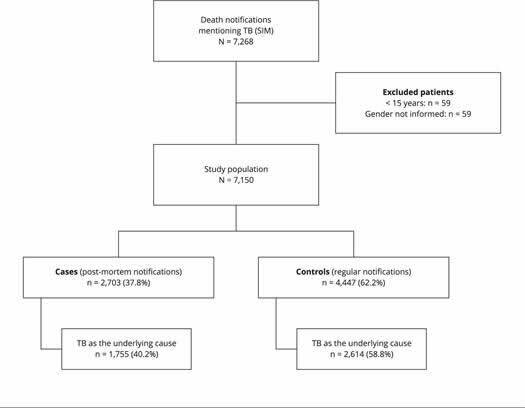
Selection of study population. Brazil, 2014.

SIM: Brazilian Mortality Information System; TB: tuberculosis.

In total, 7,268 (100%) deaths mentioning TB were reported in 2014 and 2015 (Figure 1). Of these, 118 records were excluded because they were from patients under 15 years of age and/or without gender information (Figure 1). Among the study patients, 4,447 (62.2%) were considered controls (regular disease notifications) for representing true pairs between the SINAN-TB and SIM databases, and the remaining 2,703 unpaired patients (37.8%) were considered cases (post-mortem notifications).

Post-mortem notifications include 2,506 (93%) cases reported in SIM and 197 (7%) in SINAN-TB as post-mortem cases. Among the post-mortem notification cases, 40.2% had TB as the underlying cause.

The study population of cases and controls was characterized by being mostly male (62.8%), aged 40 years or older (44.2%), black or brown (61.3%), residents in large cities (64.9%) with a high M-HDI (64.8%), low poverty rate (64.8%), located in the Southeast (60.8%) and Northeast (59.8%) regions (Table 1).

**Table 1:** Characteristics of patients and municipalities for tuberculosis (TB) cases that progressed to death according to notification status, Brazil, 2014.

Characteristics	Total deaths mentioning TB			TB as the underlying cause		
	Notification status			Notification status		
	Regular	Post-mortem	Total *	Regular	Post-mortem	Total *
	n (%)	n (%)	n	n (%)	n (%)	n
Of individuals						
Sex						
Male	3,315 (62.8)	1,963 (37.2)	5,278	1,976 (60.5)	1,290 (39.5)	3,266
Female	1,132 (60.5)	740 (39.5)	1,872	638 (57.8)	465 (42.2)	1,103
Age (complete years)						
< 20	48 (63.2)	28 (36.8)	76	29 (60.4)	19 (39.6)	48
20-39	1,239 (66.6)	662 (33.4)	1,861	516 (62.0)	317 (38.0)	833
40-59	1,877 (63.5)	1,080 (36.5)	2,957	1,090 (61.7)	676 (38.3)	1,766
60 or more	1,283 (56.9)	973 (43.1)	2,256	979 (56.8)	743 (43.2)	1,722
Educational level (years of study)						
None	506 (54.8)	418 (45.2)	924	384 (55.3)	311 (44.7)	695
1-11	2,860 (63.2)	1,666 (36.8)	4,526	1,623 (61.0)	1,039 (39.0)	2,662
> 12	140 (71.4)	56 (28.6)	196	63 (67.7)	30 (32.3)	93
Ignored	941 (62.6)	563 (37.4)	1,504	544 (59.2)	375 (40.8)	919
Race/Color						
White and yellow	1,542 (66.3)	884 (36.4)	2,426	866 (60.4)	567 (39.6)	1,433
Balck and brown	2,683 (61.4)	1,687 (38.6)	4,370	1,610 (59.4)	1,101 (40.6)	2,711
Indigenous	29 (56.9)	22 (43.1)	51	19 (51.4)	18 (48.5)	37
Ignored	193 (63.7)	110 (36.3)	303	119 (63.3)	69 (36.7)	188
Of municipalities						
M-HDI (0-1)						
Low: < 0.6	281 (52.2)	257 (47.8)	538	203 (51.4)	192 (48.6)	395
Medium: 0.6-0.7	710 (56.4)	548 (43.6)	1,258	458 (55.1)	373 (44.9)	831
High: > 0.7	3,439 (64.8)	1,869 (35.2)	5,308	1,941 (62.4)	1,169 (37.6)	3,110
Ignored	17 (37.0)	29 (63.0)	46	12 (36.4)	21 (63.6)	33
Percentage of poverty						
Low: < 10	2,491 (64.8)	1,354 (35.2)	3,845	1,356 (62.0)	831 (38.0)	2,187
Medium: 10-20	1,212 (63.5)	698 (36.5)	1,910	745 (62.5)	448 (37.5)	1,193
High: > > 20-45	457 (55.0)	374 (45.0)	831	310 (53.4)	271 (46.6)	581
Very high: > 45	270 (52.1)	248 (47.9)	518	191 (50.9)	184 (49.1)	375
Ignored	17 (37.0)	29 (63.0)	46	12 (36.4)	21 (63.6)	33
Region						
North	385 (55.6)	307 (44.4)	692	190 (47.7)	208 (52.3)	398
Northeast	1,252 (59.9)	840 (40.1)	2,092	861 (60.1)	572 (39.9)	1,433
Southeast	1,911 (60.8)	1,233 (39.2)	3,144	1,099 (57.4)	815 (42.6)	1,914
South	700 (78.0)	198 (22.0)	898	338 (79.5)	87 (20.5)	425
Central-West	199 (61.4)	125 (38.6)	324	126 (63.3)	73 (36.7)	199
Size of population (1,000 habitants)						
Small: < 20	534 (58.8)	375 (41.2)	909	355 (57.0)	268 (43.0)	623
Medium: 20-100	817 (56.2)	638 (43.8)	1,455	534 (56.0)	420 (44.0)	954
Large: > 100	3,079 (65.0)	1,661 (35.0)	4,740	1,713 (62.1)	1,046 (37.9)	2,759
Ignored	17 (37.0)	29 (63.0)	46	12 (36.4)	21 (63.6)	33
Municipalities						
Rio de Janeiro	454 (60.7)	294 (39.3)	748	247 (55.4)	199 (44.6)	446
São Paulo	314 (61.1)	200 (38.9)	514	184 (56.1)	144 (43.9)	328
Porto Alegre	167 (93.3)	12 (6.7)	179	66 (100.0)	0 (0.0)	66
Recife	143 (80.8)	34 (19.2)	177	96 (82.1)	21 (17.9)	117
Manaus	122 (74.4)	42 (25.6)	164	54 (66.7)	27 (33.3)	81
Salvador	90 (54.9)	74 (45.1)	164	57 (57.0)	43 (43.0)	100
Fortaleza	98 (65.3)	52 (34.7)	150	58 (69.1)	26 (30.9)	84
Belém	76 (52.4)	69 (47.6)	145	33 (41.3)	47 (58.7)	80
Other capitals	316 (65.2)	169 (34.8)	485	190 (66.4)	96 (33.6)	286
Other municipalities (other than capitals)	2,667 (60.3)	1,757 (39.7)	4,424	1,629 (58.6)	1,152 (41.4)	2,781
Total	4,447 (62.2)	2,703 (37.8)	7,150	2,614 (59.8)	1,755 (40.1)	4,369

M-HDI: Municipal Human Development Index.

* 100% and refer to total amount of the column, all others refer to percentage of the line.

The following groups presented higher proportions of post-mortem notifications and were higher than the general proportion of the study population (37.8%): female patients (39.5%), elderly aged 60 or older (43.1%), no schooling (45.2%), indigenous people (43.1%), residents in municipalities with low (47.7%) or medium M-HDI (43.5%), with high poverty rate (45.01%) or very high poverty rate (47.8%), mainly located in the North (44.3%), Northeast (40.1%) and Southeast (39.2%) regions, with medium (43.8%) or small population (41.2%) - such as the capitals Belém, Pará State (47.6%), Salvador, Bahia State (45.1%), Rio de Janeiro (39.30%), and other municipalities that are not capitals (39.7%) (Table 1).

Deaths with TB as the underlying cause had a similar death pattern to those cases that mentioned TB as underlying or associated cause, but the proportions of post-mortem notification were also significant among young people under 20 years of age (39.6%), among the elderly (43.2%), residents in the North (52.3%) and Southeast (42.6%) regions, and in the capital of São Paulo (43.9% of notifications) (Table 1).

After TB (61.1%), AIDS was the second most frequently mentioned disease as the underlying cause of death in the study population (25.4%), followed by neoplasms and circulatory and respiratory disorders, which accounted for less than 3% in total deaths (data not displayed).

A lower proportion of post-mortem notifications was observed among people who received medical care for the disease that caused death (3%) when compared to those who did not (18%) receive this type of care. Patients whose DC had no information about the type of health facility that assisted them and whether or not they received medical care for the disease that caused death had high proportions of post-mortem notification of TB, 90.8% and 63.3%, respectively (Table 2).

**Table 2:** Characteristics of health services, by type, primary health care coverage, and medical care provided to patients of tuberculosis (TB) that progressed to death, according to notification status. Brazil, 2014.

Characteristics	Total deaths mentioning TB			TB as the underlying cause		
	Notification status			Notification status		
	Regular	Post-mortem	Total	Regular	Post-mortem	Total
	n (%)	n (%)	n	n (%)	n (%)	n
Type of service reporting death						
Public	3,251 (67.30)	1,579 (32.70)	4,830	1,914 (65.40)	1,011 (34.60)	2,925
Private	291 (65.70)	153 (34.30)	444	157 (64.90)	85 (35.10)	242
Non-profit	849 (66.00)	437 (34.00)	1,288	501 (65.80)	261 (34.20)	762
Ignored	54 (9.20)	534 (90.80)	588	42 (9.50)	398 (90.50)	440
FSH coverage						
Low: < 50%	2,528 (62.80)	1,499 (37.20)	4,027	1,384 (59.30)	952 (40.70)	2,336
Medium: 50%-75%	820 (63.30)	476 (36.70)	1,296	500 (60.70)	324 (39.30)	824
High: > 75%	1,099 (60.20)	728 (39.80)	1,827	730 (60.60)	479 (39.60)	1,209
Primary health care coverage						
Low: < 50%	844 (57.60)	621 (42.70)	1,465	475 (54.50)	397 (45.50)	872
Medium: 50%-75%	2,158 (65.50)	1,137 (34.50)	3,295	1,198 (61.80)	739 (38.20)	1,937
High: > 75%	1,445 (60.50)	945 (39.50)	2,390	941 (60.30)	619 (39.70)	1,560
Medical care *						
Yes	2,805 (97.00)	86 (3.00)	2,891	1,538 (95.80	68 (4.20)	1,606
No	146 (82.00)	32 (18.00)	178	109 (82.60)	23 (17.40)	132
Ignored	1,496 (36.70)	2,585 (63.30)	4,081	967 (36.70)	1,664 (63.30)	2,631
Total	4,447 (62.2)	2,703 (37.8)	7,150	2,614 (59.8)	1,755 (40.2)	4,369

FHS: Family Health Strategy.

* Medical care for the disease that progressed to death, as indicated on the Death Certificate.

Table 3 shows the crude and intermediate analyses of the different models adjusted according to hierarchical levels. Table 4 shows the results of the multivariate analysis in the final model. A gradient shows increased chance of post-mortem notification of TB as the age increases, especially among people over 60 years old (OR = 1.43; 95%CI: 1.26-1.63; p < 0.001). People with less than 12 years of school education, particularly those with no schooling (OR = 1.67; 95%CI: 1.18-2.37; p = 0.003), were more likely to present this type of notification than those with more schooling years. People living in municipalities with low M-HDI (OR = 1.37; 95%CI: 1.05-1.79; p = 0.018), when compared to those living in municipalities with high M-HDI and mid-sized cities (OR = 1.26; 95%CI: 1.08-1.47; p = 0.003), and when compared to large cities and from other regions of Brazil other than the South also had a greater chance of presenting post-mortem notification. People living in Porto Alegre (Rio Grande do Sul State), Recife (Pernambuco State), and Manaus (Amazonas State) had lower chances of post-mortem notification, and those living in all other municipalities analyzed individually (Fortaleza [Ceará State], São Paulo, Rio de Janeiro, Salvador, and Belém) or together (other capitals and other municipalities) presented significantly higher chances (p < 0.001) of post-mortem notification when compared to the city of Porto Alegre (Table 4).

**Table 3:** Crude and multivariate analyses (partial analyses by hierarchical levels) of the association between selected variables and the chance of post-mortem notification of tuberculosis (TB). Brazil, 2014.

Variable (reference category)	Crude analysis			Adjusted analysis (partial)		
	OR	95%CI	p-value	OR	95%CI	p-value
Model 1						
Individual characteristics						
Sex (male)						
Female	1.10	0.99-1.23	0.073			
Age (< 40 years) [complete years]						
40-59	1.14	1.01-1.28	0.034	1.12	1.00-1.27	0.049
≥ 60	1.50	1.32-1.70	< 0.001	1.44	1.27-1.64	< 0.001
Educational level (≥ 12 years) [years of study]						
1-11	1.45	1.06-1.99	0.020	1.48	1.07-2.03	0.015
None	2.06	1.47-2.88	< 0.001	1.95	1.39-2.73	< 0.001
Ignored	1.49	1.07-2.07	0.016	1.50	1.08-2.09	0.014
Race/Color (ignored)						
White and yeallow	1.00	0.78-1.29	0.960			
Balck and brown	1.10	0.86-1.40	0.420			
Indigenous	1.33	0.72-2.42	0.350			
Model 2						
Municipalities characteristics						
M-HDI (high: > 0.7)						
Medium: 0.6-0.7	1.42	1.25-1.60	< 0.001	1.17	0.97-1.40	0.085
Low: < 0.6	1.76	1.48-2.09	< 0.001	1.38	1.06-1.80	0.014
Percentage of poverty (low: < 10)						
Medium: 10-20	1.05	0.94-1.18	0.321			
High: 20-45	1.50	1.29-1.75	< 0.001			
Very high: > 45	1.77	1.40-2.12	< 0.001			
Population size (large)						
Medium	1.44	1.28-1.63	< 0.001	1.27	0.97-1.40	0.002
Small	1.35	1.17-1.56	< 0.001	1.38	1.06-1.80	0.344
Region (Soth)						
Central-West	2.22	1.68-2.91	< 0.001	1.08	1.36-2.43	< 0.001
Southeast	2.28	1.91-2.71	< 0.001	1.89	1.55-2.29	< 0.001
Northeast	2.37	1.98-2.84	< 0.001	1.81	1.45-2.27	< 0.001
North	2.81	2.26-3.50	< 0.001	2.52	1.90-3.33	< 0.001
Municipalities (Porto Alegre)						
Recife	3.30	1.65-6.62	0.001	1.81	0.87-3.77	0.108
Manaus	4.79	2.42-9.48	< 0.001	1.90	0.90-3.97	0.088
Fortaleza	7.38	3.75-14.50	< 0.001	4.06	1.99-8.27	< 0.001
São Paulo	8.86	4.80-16.34	< 0.001	4.22	2.21-8.04	< 0.001
Rio de Janeiro	9.01	4.92-16.48	< 0.001	4.68	2.46-8.90	< 0.001
Salvador	11.44	5.90-22.17	< 0.001	4.76	2.52-8.98	< 0.001
Belém	12.63	6.46-24.69	< 0.001	4.31	2.33-7.97	< 0.001
Other capitals *	7.44	4.02-13.76	< 0.001	6.29	3.13-12.6	< 0.001
Other municipalities (other than capitals)	9.16	5.08-16.52	< 0.001	5.01	2.42-10.3	< 0.001
Model 3						
Causes of death						
Underlying causes (tuberculosis)						
AIDS	0.61	0.54-0.69	< 0.001	0.65	0.56-0.71	< 0.001
Malignant neoplasms	0.63	0.45-0.87	0.006	0.36	0.45-0.87	0.006
Viral hepatitis	0.81	0.29-2.20	0.683	0.92	0.33-2.53	0.881
Diabetes	0.82	0.44-1.51	0.530	0.79	0.42-1.48	0.474
Respiratory diseases	1.51	1.07-2.12	0.018	1.47	1.04-2.07	0.028
Hypertension	1.95	1.01-3.75	0.044	2.09	1.08-4.03	0.027
Mental and behavioral disorders **	2.06	1.12-3.80	0.019	2.05	1.11-3.78	0.020
Malnutrition	6.45	1.83-22.68	0.004	6.38	1.81-22.45	0.004
Other	1.14	0.94-1.37	0.170	1.14	0.94-1.37	0.171
Model 4						
Health servuices characteristics						
Type of service (public)						
Private	1.08	0.88-1.32	0.488			
Non-profit	1.05	0.92-1.20	0.401			
Ignored	20.36	15.29-27.10	< 0.000			
FHS coverage (medium: 50%-75%)						
Low: < 50%	1.02	0.89-1.16	0.748			
High: > 75%	1.14	0.98-1.32	0.078			
Primary health care coverage (medium: 50%-75%)						
Low: < 50%	1.39	1.23-1.58	< 0.001	0.99	0.86-1.14	0.978
High: > 75%	1.24	1.11-1.38	< 0.001	1.42	1.20-1.68	< 0.001
Medical care (yes)						
No	7.14	4.61-11.08	< 0.001	4.97	3.08-8.02	< 0.001
Ignored	56.35	45.05-70.49	< 0.001	49.45	39.43-62.02	< 0.001

95%CI: 95% confidence interval; FHS: Family Health Strategy; M-HDI: Municipal Human Development Index; OR: odds ratio.

* Other municipalities that are capitals of states;

** Mental and behavioral disorders due to the use of psychoactive substances.

**Table 4:** Multivariate analysis of the association between selected variables and the chance of post-mortem notification of tuberculosis (TB). Brazil, 2014.

Variable (referebce category)	Adjusted analysis		
	OR	95%CI	p-value
Model 5: adjusted according to variables of the same hierarchical level			
Individual characteristics			
Age (< 40 years) [complete years]			
40-59	1.15	1.01-1.29	0.030
≥ 60	1.43	1.26-1.63	< 0.001
Educational level (≥ 12 years) [years of study]			
1-11	1.45	1.05-2.00	0.023
None	1.67	1.18-2.37	0.003
Ignored	1.39	0.99-1.94	0.052
Municipalities characteristics			
M-HDI (high: > 0.7)			
Medium: 0.6-0.7	1.15	0.96-1.39	0.119
Low: < 0.6	1.37	1.05-1.79	0.018
Population size (large)			
Medium	1.26	1.08-1.47	0.003
Small	1.05	0.86-1.28	0.609
Region (South)			
Central-West	1.76	1.31-2.35	< 0.001
Southeast	1.85	1.53-2.25	< 0.001
Northeast	1.75	1.39-2.19	< 0.001
North	2.42	1.82-3.21	< 0.001
Municipalities (Porto Alegre)			
Recife	1.84	0.88-3.82	0.102
Manaus	1.99	0.95-4.18	0.068
Fortaleza	4.14	2.03-8.46	< 0.001
São Paulo	4.32	2.26-8.24	< 0.001
Rio de Janeiro	4.76	2.50-9.06	< 0.001
Salvador	4.86	2.57-9.18	< 0.001
Belém	4.41	2.38-8.16	< 0.001
Other capitals *	6.47	3.21-13.04	< 0.001
Other municipalities (other than capitals)	5.29	2.55-10.98	< 0.001
Modelo 6: adjusted according to variables of model 5			
Causes of death			
Causes of death			
Underlying causes (tuberculosis)	0.75	0.65-0.85	< 0.001
AIDS	0.62	0.44-0.87	0.005
Malignant neoplasms	1.13	0.39-3.28	0.811
Viral hepatitis	0.76	0.40-1.43	0.404
Diabetes	1.54	1.08-2.18	0.015
Respiratory diseases	1.77	0.91-3.43	0.090
Hypertension	2.36	1.26-4.42	0.007
Mental and behavioral disorders **	5.54	1.57-19.54	0.008
Malnutrition	1.20	0.99-1.46	0.054
Model 7: adjusted according to variables of model 6			
Health services characteristics			
Primary health care coverage (medium: 50%-75%)			
Low: < 50%	1.18	0.95-1.47	0.122
High: > 75%	0.79	0.65-0.96	0.022
Medical care (yes)			
No	6.86	4.38-10.74	< 0.001
Ignored	58.55	46.55-73.65	< 0.001

95%CI: 95% confidence interval; M-HDI: Municipal Human Development Index; OR: odds ratio.

* Other municipalities that are capitals of states;

** Mental and behavioral disorders due to the use of psychoactive substances.

People with HIV (OR = 0.75) or neoplasms (OR = 0.62), when these disorders are the underlying cause of death, are significantly associated with lower chances of post-mortem notification of TB, when compared to people who had TB as the main cause of death. On the other hand, people who had other respiratory diseases (OR = 1.54; 95%CI: 1.08-2.18; p = 0.015), mental and behavioral disorders (OR = 2.36; 95%CI: 1.26-4.42; p = 0.007), and malnutrition (OR = 5.54; 95%CI: 1.57-19.54; p = 0.008) were more likely to present post-mortem notification of TB than people with TB as the underlying cause (Table 3). In this analysis, hypertension as the underlying cause also showed an increased chance of post-mortem notification (OR = 1.77) when compared to TB as the underlying cause, but it was not statistically significant (p = 0.090).

Residents of municipalities with high primary health care coverage also had a lower chance of post-mortem notification of TB (OR = 0.79; 95%CI: 0.65-0.96; p = 0.022) when compared to residents of municipalities with low and medium primary health care coverage. In addition, people who did not have medical care for the disease that caused death were more likely to have post-mortem notifications of TB (OR = 6.86; 95%CI: 4.38-10.74; p < 0.001) than people they had. Also, the lack of information about the provision of care presented a high chance of having post-mortem notification of TB after adjustment (OR = 58.55; 95%CI: 46.55-73.65; p < 0.001) (Table 4).

## Discussion

This study showed that, among the deaths from TB reported in Brazil in 2014, 38% had TB notification only at death (post-mortem notification). People who were older, with less education, who lived in cities with the worst municipal human development indicators (low M-HDI), with low coverage of primary health care, and without information of having received medical support for TB were more likely to present post-mortem notification of TB.

Post-mortem notification of TB can be a consequence of different scenarios: (i) underdetection of the case until death, (ii) detection of the case, but without proper treatment and notification, or (iii) detection and treatment, but without the due notification to SINAN-TB (exclusively underreporting). In all these three scenarios, the development of suitable and timely TB surveillance actions is compromised ^12^.

No difference was observed in the chance of post-mortem notification among female patients when compared to male patients (OR = 1.10; 95%CI: 0.99-1.23). In a study conducted in João Pessoa (Paraíba State), which analyzed the underreporting of TB cases between 2001 and 2010, female patients presented a higher chance of TB underreporting when compared to male patients ^13^. One hypothesis of this finding would be higher sensitivity of Koch’s bacillus detection in sputum samples from male patients ^13^ and easier sputum extraction from the pectoral muscles of male patients. On the other hand, men tend to seek health services less often when compared to women ^14^ ^,^ ^15^. Then, competing factors in opposite directions, such as fewer visits to the service, could neutralize the protective effect of the male gender and justify the findings of our study of non-association between sex and the chance of post-mortem notification of TB.

People aged 60 years or older presented higher chances of notification only at death, in agreement with a study conducted in a municipality in the state of Rio de Janeiro ^16^. In addition, elderly people tend to present higher proportions of poorly defined underlying causes or with “unspecified” codes ^17^, which can be explained by the coexistence of multiple chronic diseases with older age, resulting in difficult diagnosis and masked TB.

A higher chance of post-mortem notification was observed among residents of municipalities with low M-HDI. A study conducted by the WHO, which assessed the trend of TB and its determinants in 134 countries ^18^, found a stronger decline in the incidence of TB in countries with higher M-HDI. As one of well-known socioeconomic factors that increase the vulnerability of subjects to TB ^19^, low educational level presented a higher chance of post-mortem notification. It can be explained by the unequal access to information and health resources - including TB diagnosis and treatment - among people with low levels of education, representing a group with high underreporting of TB cases ^20^.

TB perpetuation in impoverished regions and municipalities suggests vulnerable population; weaknesses in disease prevention, control, and surveillance network; and a higher probability of underdetection and underreporting of cases and contacts. In an ecological study, Pelissari et al. ^21^ identified scenarios according to socioeconomic, epidemiological, and operational variables. The authors describe an inverse association between selected operational indicators - related to good performance of disease surveillance - and the TB incidence rate, particularly in the combined rate of low socioeconomic status of the municipalities.

Our study also identified that people who did not receive care for the disease that caused death had a higher chance to present post-mortem notification than others. This fact reinforces the idea that such group is comprised of subjects who are excluded from the health care system and who, most likely, did not have a chance to access timely and adequate diagnosis or treatment of TB or the underlying cause of death. Also, what Mendes ^22^ warns about the organizational and operational contexts of the health system in Brazil must be considered. Historically, care has been provided in a fragmented, reactive, and episodic manner in many settings. This result leads to reflections on the challenges in the management of TB treatment, which, as a chronic condition, requires organized services and an organized network of continuous and comprehensive care.

In this study, having malnutrition as the underlying cause of death was associated with a higher chance of post-mortem notification of TB. Malnutrition as the underlying cause of death is a marker of significant physical weakness and, perhaps, of low socioeconomic status and lack of access to quality health services, reinforcing the assumption that it is a population of high social vulnerability ^23^. On the other hand, other respiratory diseases, mental and behavioral disorders, and even malnutrition, are issues that can determine multiple contacts with health professionals and services ^24^ ^,^ ^25^. For patients with actual contacts with medical services, a higher chance of post-mortem notification of TB may be the result of lower TB diagnosis during visits to health services due to other issues, leading to death without adequate previous TB detection. Reflections on comprehensive care in this hypothetical context are required.

On the other hand, people with AIDS or neoplasms as the underlying cause of death were less likely to present post-mortem notification of TB when compared to the group that had TB as the underlying cause of death. A study conducted by Santos et al. ^13^ reported a lower proportion (29%) of underreported TB in SINAN-AIDS when compared to SIM (39%). A high sensitivity of the health system may detect TB among people with AIDS, which may partly explain this fact, but not true for other diseases, as mentioned above. Also, people with AIDS demand multiple contacts with specialized health services and professionals due to treatment specificities and follow-up exams, which may partially explain these findings and perhaps be also true for people with neoplasm.

A lower chance of presenting post-mortem notification of TB was observed among residents of municipalities with high primary health care coverage when compared to those living in municipalities with medium and low coverage. Studies have described that broad primary health care coverage, especially in the FHS, has a relevant impact on several health outcomes, such as reduction of infant mortality and risk of having cardiovascular diseases ^26^ ^,^ ^27^. Priemary health care represents a structuring axis of the SUS, a front door to health services with roles of resolution, coordination, and accountability ^22^, and due to its characteristics of offering comprehensive care, it is expected to facilitate the prevention, detection, and follow-up of people with TB, who in theory can be benefited in this scenario. However, challenges persist in the decentralization of TB control actions in primary health care, as described by Wysocki et al. ^28^, who highlighted weaknesses of different natures, such as poor engagement of professionals in control actions, centralized verticalization of control actions in primary health care, staff turnover, gaps in professional training, lack of progress in the articulation between health care facilities, among others.

In addition to the characteristics of primary health care mentioned above, Pelissari et al. ^21^ studied the association between the supply of primary health care and TB incidence in Brazil. Using adjusted models, the study estimated that a 10% increase in primary health care coverage in the municipalities is associated with a 2.24% reduction in the TB incidence rate, and that TB detection was associated with different characteristics of services and actions offered in primary health care, such as the active search for TB cases. The findings of Pelissari et al. ^21^ agree with the results of our study, suggesting that the supply of services by primary health care plays an important role in TB surveillance, including prevention, diagnosis, and late treatment of TB at the local level.

In agreement with 2015 mortality rates from the Brazilian Ministry of Health, the Northeast and North regions had a higher chance of post-mortem notification of TB ^9^ ^,^ ^29^. The same finding was obtained in a study conducted from 2012 to 2014, which analyzed 14 data quality indicators, opportunity, and acceptability of TB surveillance in Brazil ^30^. The authors identified significant geographical inequalities in these indicators, suggesting priority micro-regions for surveillance improvement are predominantly located in the central-north regions of Brazil, which partially agree with our findings. It can be explained by the fact that both regions have large portions of the population subject to high social vulnerability ^9^ ^,^ ^29^.

Study limitations include those related to the use of secondary data, with inadequate filling of forms, as can be seen in some variables. Another limitation refers to possible failures of the probabilistic relationship used in this study. In our study, we considered deaths mentioning TB found exclusively in SIM as a post-mortem notification, assuming these people had no previous notification. The probabilistic relationship of databases may have had classification errors in its process. Also, the “post-mortem” field was only included in SINAN-TB in late 2014. Then, before this period, deaths with this characteristic were not properly identified in SINAN. Finally, and most importantly, it is impossible to discriminate in post-mortem notifications identified in this study cases that were underreported only from those that were underdetected - as discussed above. Anyway, according to our results, cases that are underreported only are not very relevant, since the profile found here is consistent with the profile of underserved population in Brazil with significant barriers in accessing health services.

This study is the first to identify factors associated with post-mortem notification of TB in Brazil using a database derived from a probabilistic relationship of SINAN-TB and SIM. In Brazil, death from TB - mainly pulmonary TB - is a sentinel event that provides warning of failure in care and surveillance of the disease, since diagnosis, clinical management, and effective treatment are available in primary health care ^1^. Death that mentions TB detected after death indicates fragility of this care network, compromising the prevention and control of new cases and interruption of TB chain of transmission in the community. Therefore, it should be prioritized among disease control actions. The results suggest the creation of a field of study to address new research questions, and provide elements to support a portion of TB cases that are invisible to the disease surveillance and care system.
